# Neurologic and psychiatric disorders as risk factors following hip arthroplasty: results from the German arthroplasty registry

**DOI:** 10.1007/s00264-025-06593-2

**Published:** 2025-06-26

**Authors:** Nele Wagener, Alexander Grimberg, Yinan Wu, Sebastian Hardt, Carsten Perka

**Affiliations:** 1 Center for Musculoskeletal Surgery, Department of Orthopaedic Surgery, Charité Universitätsmedizin Berlin, corporate member of Freie Universität Berlin and Humboldt-Universität zu Berlin, Charitéplatz 1, 10117 Berlin, Germany; 2 Endoprothesenregister Deutschland (EPRD), 10623 Berlin, Germany

**Keywords:** Hip arthroplasty, Neurologic disorders, Psychiatric disorders, Causes of revision, Mortality, Registry study

## Abstract

**Purpose:**

We investigated whether neurologic and psychiatric disorders (ICD-10 F00–F99, G00–G99) increase postoperative complications and mortality after hip arthroplasty and identified subgroups with distinct complication patterns, including dislocations, loosening, fractures, and elevated mortality.

**Methods:**

We analyzed 190,340 primary cementless hip arthroplasties from the German Arthroplasty Registry (2012–2024). Patients with relevant diagnoses were compared to matched controls (1:1 Mahalanobis distance) across subgroups F00–F99 and G00–G99, adjusting for age, sex, BMI, Elixhauser Index, and arthroplasty type. Primary endpoints were implant survival (time to revision) and all-cause mortality over up to eight years. Revision causes including periprosthetic fracture, infection, dislocation, loosening, and others were systematically recorded.

**Results:**

Most subgroups showed significantly higher revision rates (*p* < 0.0001 for F00–F09, F10–F19, F30–F39, G20–G26, G40–G47, G60–G64). Mortality was also significantly higher (*p* < 0.0001 for F00–F09, F10–F19, F30–F39). Schizophrenia (F20–F29) increased revision (*p* < 0.0001) and mortality (*p* < 0.0001). Organic mental disorders (F00–F09) showed markedly elevated revision and mortality rates, with more frequent dislocations and fractures (*p* < 0.0001). Extrapyramidal disorders (G20–G26) mainly increased dislocation risk (*p* = 0.00032), while degenerative diseases (G30–G32) raised mortality (*p* < 0.0001). Episodic/paroxysmal disorders (G40–G47) increased loosening (*p* = 0.0041) and revision (*p* < 0.0001). Polyneuropathies (G60–G64) were linked to joint instability and dislocations (*p* = 0.0008).

**Conclusion:**

Neurologic and psychiatric disorders significantly elevate revision and mortality risks following hip arthroplasty. Subgroup-specific vulnerabilities, dislocations/fractures (F00–F09), high complication and mortality (F10–F19), and joint instability (G60–G64), highlight the need for individualized perioperative strategies and close postoperative monitoring to improve outcomes.

## Introduction

Neurological and psychiatric disorders are widespread in the general population and are of increasing significance with advancing age [1]. These disorders are particularly prevalent in older adulthood and can influence the postoperative course following orthopedic interventions [1, 2]. In this context, it is important to note that patients with cognitive or mental impairments may have an increased risk of complications; for instance, movement disorders can make mobilization more difficult, while affective or cognitive disorders can impair compliance with rehabilitation measures [3].

Although comorbidities have been generally well investigated as risk factors for revision surgeries and mortality following total hip arthroplasty (THA), there are currently only limited data that specifically examine the differentiated influence of neurological and psychiatric diagnoses (according to ICD-F and G chapters) [4]. Against this background, the present registry-based study employed a 1:1-matched study design to investigate whether and to what extent defined neurological or psychiatric pre-existing conditions (e.g., movement disorders, dementia, psychotropic substance use disorders, or affective disorders) increase the risk of revision surgeries and mortality after THA. The aim was to conduct targeted subgroup analyses and thus fill gaps that remain in the current literature due to the frequent combined evaluation of ICD-F and G diagnoses [5]. Therefore, the central hypothesis of this work is: Do specific neurological or psychiatric pre-existing conditions significantly influence revision rates and mortality after hip arthroplasty over the long term?

## Materials and methods

### Data source

We used data from the German Arthroplasty Registry (EPRD) for the period from November 2012 to March 30, 2024 [5]. In total, 684,489 hip endoprostheses were documented, of which 455,965 were elective or hemiprostheses. Among these, 89,852 cases had at least one F and/or G diagnosis code. For the 1:1 matching, 95,170 cases were included in each group, that is, 95,170 in the F/G group and 95,170 in the control group with replacement, resulting in a total of 190,340 cases (Table 1a and 1b). Documentation was based on (1) billing data from statutory health insurance providers, (2) a product database from implant manufacturers, and (3) electronic case reports from participating hospitals. By linking these data with insurance records, patient follow-up was ensured. Coding followed OPS 301 and ICD-10 guidelines.

To ensure a valid data basis, all records lacking information on implant type, indication diagnosis, or follow-up were excluded. For the variables BMI and reasons for revision, some entries were not further specified and were assigned to their own category (“not specified/unknown”). Ethical approval from the University of Kiel (D 473/11) had been obtained. Potential limitations arising from the registry data are discussed in detail in the Discussion section.

**Table 1a:** Baseline characteristics after 1:1 matching for patients without and with psychiatric disorders (ICD-10 F00–F09; F10-F19; F20-F29; F30-F39)

	F00-F09			F10-F19			F20-F29			F30-F39		
Characteristic	Without psychiatric *N* = 7,166	With psychiatric *N* = 7,166	Difference	Without psychiatric *N* = 9,625	With psychiatric *N* = 9,625	Difference	Without psychiatric *N* = 981	With psychiatric *N* = 981	Difference	Without psychiatric *N* = 21,648	With psychiatric *N* = 21,648	Difference
Sex			0,03			0			0			0
Female	4,108.0 (57.3%)	4,015.0 (56.0%)		4,569.0 (47.5%)	4,565.0 (47.4%)		644.0 (65.6%)	643.0 (65.5%)		16,195.0 (74.8%)	16,195.0 (74.8%)	
Male	3,058.0 (42.7%)	3,151.0 (44.0%)		5,056.0 (52.5%)	5,060.0 (52.6%)		337.0 (34.4%)	338.0 (34.5%)		5,453.0 (25.2%)	5,453.0 (25.2%)	
Age	79.6 (8.5)	80.4 (8.3)	-0,1	60.1 (10.3)	59.8 (10.3)	0,02	62.7 (11.5)	62.6 (11.6)	0,02	66.2 (10.5)	65.8 (10.5)	0,04
BMI			0,02			0			0			0
Underweight [< 18.5]	163.0 (2.3%)	168.0 (2.3%)		106.0 (1.1%)	106.0 (1.1%)		18.0 (1.8%)	18.0 (1.8%)		137.0 (0.6%)	137.0 (0.6%)	
Normal [18.5-24.99]	2,360.0 (32.9%)	2,366.0 (33.0%)		2,074.0 (21.5%)	2,074.0 (21.5%)		163.0 (16.6%)	163.0 (16.6%)		3,822.0 (17.7%)	3,822.0 (17.7%)	
Pre-obese[ 25.0-29.99]	1,869.0 (26.1%)	1,864.0 (26.0%)		2,680.0 (27.8%)	2,680.0 (27.8%)		231.0 (23.5%)	231.0 (23.5%)		5,816.0 (26.9%)	5,816.0 (26.9%)	
Obese 1 [30.0-34.99]	778.0 (10.9%)	737.0 (10.3%)		1,671.0 (17.4%)	1,671.0 (17.4%)		187.0 (19.1%)	187.0 (19.1%)		4,235.0 (19.6%)	4,235.0 (19.6%)	
Obese 2 [35.0-39.99]	199.0 (2.8%)	187.0 (2.6%)		624.0 (6.5%)	624.0 (6.5%)		85.0 (8.7%)	85.0 (8.7%)		1,930.0 (8.9%)	1,930.0 (8.9%)	
Obese 3 [ > = 40]	67.0 (0.9%)	67.0 (0.9%)		290.0 (3.0%)	290.0 (3.0%)		53.0 (5.4%)	53.0 (5.4%)		1,006.0 (4.6%)	1,006.0 (4.6%)	
Not specified	1,730.0 (24.1%)	1,777.0 (24.8%)		2,180.0 (22.6%)	2,180.0 (22.6%)		244.0 (24.9%)	244.0 (24.9%)		4,702.0 (21.7%)	4,702.0 (21.7%)	
Elixhauser score	6.9 (7.3)	7.5 (7.9)	-0,08	1.1 (5.0)	0.9 (5.3)	0,03	2.3 (5.7)	2.3 (5.8)	0,01	-0.4 (4.8)	-1.0 (5.1)	0,12
Type of arthroplasty			0			0			0			0
Elective THA	3,861.0 (53.9%)	3,861.0 (53.9%)		9,290.0 (96.5%)	9,290.0 (96.5%)		870.0 (88.7%)	870.0 (88.7%)		20,823.0 (96.2%)	20,823.0 (96.2%)	
Hemiarthroplasty	3,305.0 (46.1%)	3,305.0 (46.1%)		335.0 (3.5%)	335.0 (3.5%)		111.0 (11.3%)	111.0 (11.3%)		825.0 (3.8%)	825.0 (3.8%)	

Descriptive statistics of both cohorts after 1:1 matching, comparing patients without and with psychiatric pre-existing conditions across ICD-10 diagnostic groups (F00–F09: *N* = 7,166; F10–F19: *N* = 9,625; F20–F29: *N* = 981; F30–F39: *N* = 21,648 per group). Displayed are sex, age, BMI categories, and Elixhauser comorbidity score, along with corresponding *p*-values derived from Pearson’s chi-squared test or Welch’s two-sample *t*-test

**Table 1b:** Baseline characteristics after 1:1 matching for patients without and with neurological disorders (ICD-10 G20-G26; G30-G32; G40-G47; G60-G64)

	G20–G26			G30–G32			G40–G47			G60–G64		
Characteristic	Without neurological *N* = 10,173	With neurological *N* = 10,173	Difference	Without neurological *N* = 913	With neurological *N* = 913	Difference	Without neurological *N* = 38,560	With neurological *N* = 38,560	Difference	With neurological *N* = 6,104	With neurological *N* = 6,104	Difference
Sex			0			0			0			0
Female	7,313.0 (71.9%)	7,310.0 (71.9%)		559.0 (61.2%)	559.0 (61.2%)		19,742.0 (51.2%)	19,733.0 (51.2%)		3,361.0 (55.1%)	3,361.0 (55.1%)	
Male	2,860.0 (28.1%)	2,863.0 (28.1%)		354.0 (38.8%)	354.0 (38.8%)		18,818.0 (48.8%)	18,827.0 (48.8%)		2,743.0 (44.9%)	2,743.0 (44.9%)	
Age	71.4 (9.4)	71.4 (9.4)	0	80.3 (8.0)	80.4 (8.2)	-0,01	66.6 (10.1)	66.6 (10.1)	0	71.1 (9.8)	71.0 (9.9)	0
BMI			0			0			0			0
Underweight [< 18.5]	77.0 (0.8%)	77.0 (0.8%)		33.0 (3.6%)	33.0 (3.6%)		168.0 (0.4%)	168.0 (0.4%)		38.0 (0.6%)	38.0 (0.6%)	
Normal [18.5-24.99]	2,043.0 (20.1%)	2,043.0 (20.1%)		329.0 (36.0%)	329.0 (36.0%)		5,765.0 (15.0%)	5,765.0 (15.0%)		1,078.0 (17.7%)	1,078.0 (17.7%)	
Pre-obese [25.0-29.99]	2,892.0 (28.4%)	2,893.0 (28.4%)		227.0 (24.9%)	228.0 (25.0%)		10,409.0 (27.0%)	10,409.0 (27.0%)		1,742.0 (28.5%)	1,742.0 (28.5%)	
Obese 1 [30.0-34.99]	1,773.0 (17.4%)	1,772.0 (17.4%)		80.0 (8.8%)	79.0 (8.7%)		8,155.0 (21.1%)	8,155.0 (21.1%)		1,229.0 (20.1%)	1,229.0 (20.1%)	
Obese 2 [35.0-39.99]	739.0 (7.3%)	739.0 (7.3%)		12.0 (1.3%)	12.0 (1.3%)		4,443.0 (11.5%)	4,443.0 (11.5%)		490.0 (8.0%)	490.0 (8.0%)	
Obese 3 [ > = 40]	286.0 (2.8%)	286.0 (2.8%)		2.0 (0.2%)	2.0 (0.2%)		2,179.0 (5.7%)	2,179.0 (5.7%)		248.0 (4.1%)	248.0 (4.1%)	
Not specified	2,363.0 (23.2%)	2,363.0 (23.2%)		230.0 (25.2%)	230.0 (25.2%)		7,441.0 (19.3%)	7,441.0 (19.3%)		1,279.0 (21.0%)	1,279.0 (21.0%)	
Elixhauser score	3.6 (6.2)	3.6 (6.2)	0	7.5 (7.6)	7.5 (7.7)	0	1.8 (5.2)	1.8 (5.3)	0	3.4 (6.2)	3.3 (6.3)	0
Type of arthroplasty			0			0			0			0
Elective THA	9,403.0 (92.4%)	9,403.0 (92.4%)		370.0 (40.5%)	370.0 (40.5%)		37,802.0 (98.0%)	37,802.0 (98.0%)		5,745.0 (94.1%)	5,745.0 (94.1%)	
Hemiarthroplasty	770.0 (7.6%)	770.0 (7.6%)		543.0 (59.5%)	543.0 (59.5%)		758.0 (2.0%)	758.0 (2.0%)		359.0 (5.9%)	359.0 (5.9%)	

Descriptive statistics of both cohorts after 1:1 matching, comparing patients without and with neurological pre-existing conditions across ICD-10 diagnostic groups (G20–G26: *N* = 10,173; G30–G32: *N* = 913; G40–G47: *N* = 38,560; G60–G64: *N* = 6,104 per group). Displayed are sex, age, BMI categories, and Elixhauser comorbidity score, along with corresponding *p*-values derived from Pearson’s chi-squared test or Welch’s two-sample *t*-test

### Study design and patient selection

Only adult patients undergoing an elective primary hip arthroplasty were included. Exclusively cementless implants were considered to ensure a uniform cohort.

Neurological and psychiatric diagnoses were extracted using ICD 10 codes F00–F99 and G00–G99. To allow for a more differentiated analysis and reflect existing knowledge, these diagnoses were grouped into eight subgroups:

#### F-diagnoses

1. Organic, including symptomatic, mental disorders (F00–F09).
2. Mental and behavioral disorders due to psychoactive substances (F10–F19).
3. Schizophrenia, schizotypal, and delusional disorders (F20–F29).
4. Affective disorders (F30–F39).

#### G-diagnoses

5. Extrapyramidal and movement disorders (G20–G26).
6. Degenerative diseases of the nervous system (G30–G32).
7. Episodic and paroxysmal disorders (G40–G47).
8. Polyneuropathies and other disorders of the peripheral nervous system (G60–G64).

Patients with at least one of these diagnoses during the billing period were assigned to the “Neurology/Psychiatry” group; all others constituted the control group. Since the registry contains the diagnoses at least once during the inpatient stay (or in preceding quarters), assignment was based on all ICD entries.

To reduce potential confounding, we performed a 1:1 matching using Mahalanobis distance for each subgroup (F00–F99 and G00–G99), adjusting for age, sex, BMI, and the Elixhauser Comorbidity Index (ECI), type of arthorplasty. This approach yielded comparably large cohorts for further analysis in each subgroup and its corresponding control group.

### Collected parameters and endpoints

In addition to sociodemographic and clinical baseline data, comorbidities were determined using the Elixhauser Score. The primary outcome parameters were implant survival (time to revision) and mortality. Furthermore, various reasons for revision (e.g., periprosthetic fracture, infection, implant loosening, dislocation) were investigated for a possible association with neurological/psychiatric pre-existing conditions.

### Statistical analysis

Descriptive statistics were used to characterize both study cohorts after matching. Continuous variables were reported as means with standard deviations, while categorical variables were presented as frequencies and percentages. Standardized mean differences (SMDs) were calculated to assess the quality of matching; an SMD less than 0.1 was considered indicative of adequate balance between groups.

Implant survival, mortality, and reasons for revision over time were analyzed using Kaplan–Meier methods over a follow-up period of up to eight years, with 95% log–log confidence intervals. Group differences were assessed using the log-rank test, with a significance level set at *P* < 0.05. All statistical analyses were performed using R (version 4.4.2, R Foundation for Statistical Computing, Vienna, Austria).

## Results

Neurological and psychiatric disorders were associated with significantly increased revision and mortality rates following primary cementless hip arthroplasty.

**Fig. 1:**
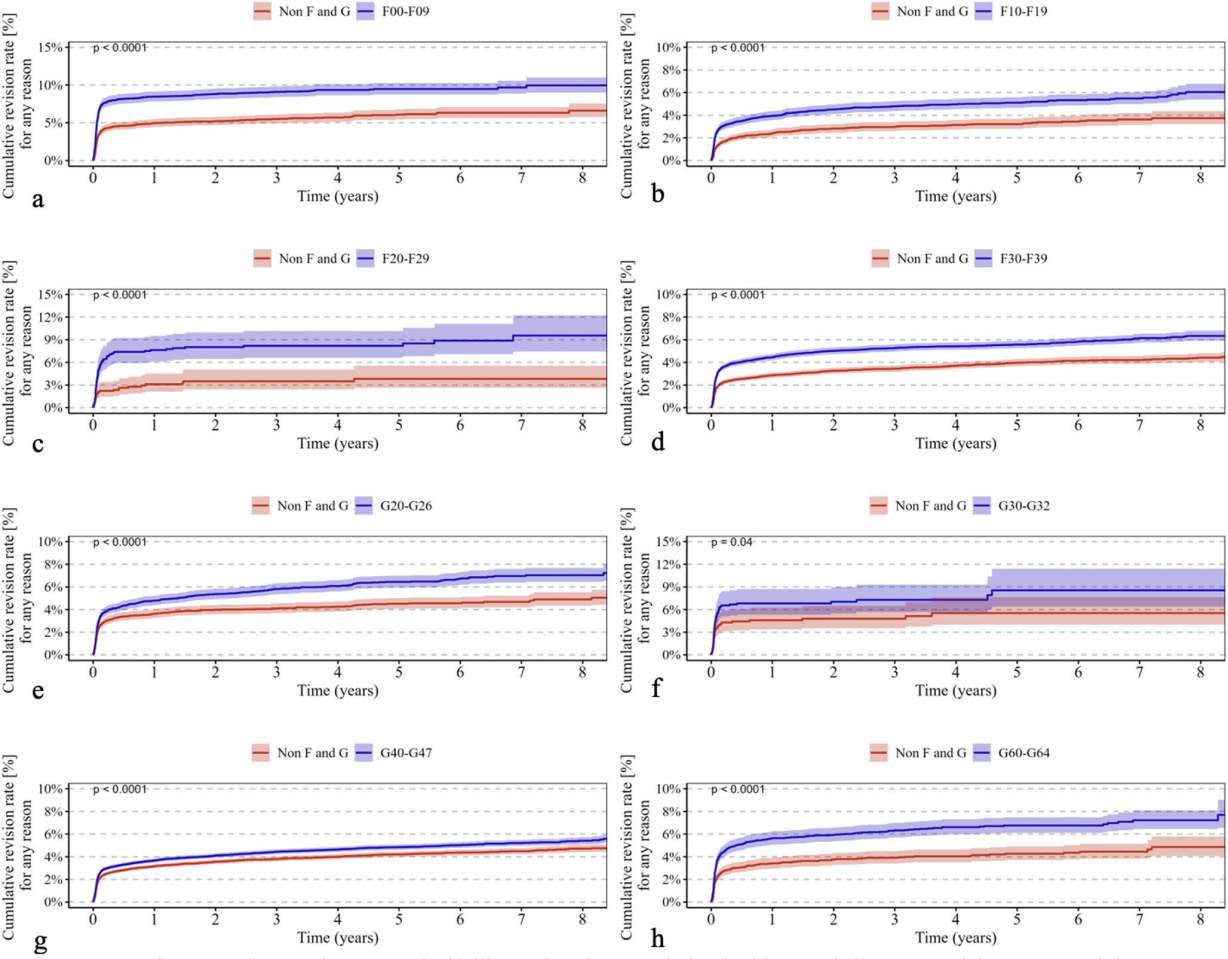
Shows Kaplan–Meier curves (**a**–**h**) illustrating the cumulative incidence of all-cause revision over an eight-year observation period in patients with the following ICD-10 diagnoses (red curves) compared with a control group (blue curves): (**a**) F00–F09 (“Organic, including symptomatic, mental disorders,” *p* < 0.0001), (**b**) F10–F19 (“Mental and Behavioral Disorders due to Psychoactive Substance Use,” *p* < 0.0001), (**c**) F20–F29 (“Schizophrenia, schizotypal, and delusional disorders,” *p* < 0.0001), (**d**) F30–F39 (“Affective Disorders,” *p* < 0.0001), (**e**) G20–G26 (“Extrapyramidal and Movement Disorders,” *p* < 0.0001), (**f**) G30–G32 (“Degenerative diseases of the nervous system,” *p* = 0.04), (**g**) G40–G47 (“Episodic and paroxysmal disorders,” *p* < 0.0001), and (**h**) G60–G64 (“Polyneuropathies and other disorders of the peripheral nervous system,” *p* < 0.0001). The *p*-values indicate the significance of between-group differences

**Fig. 2:**
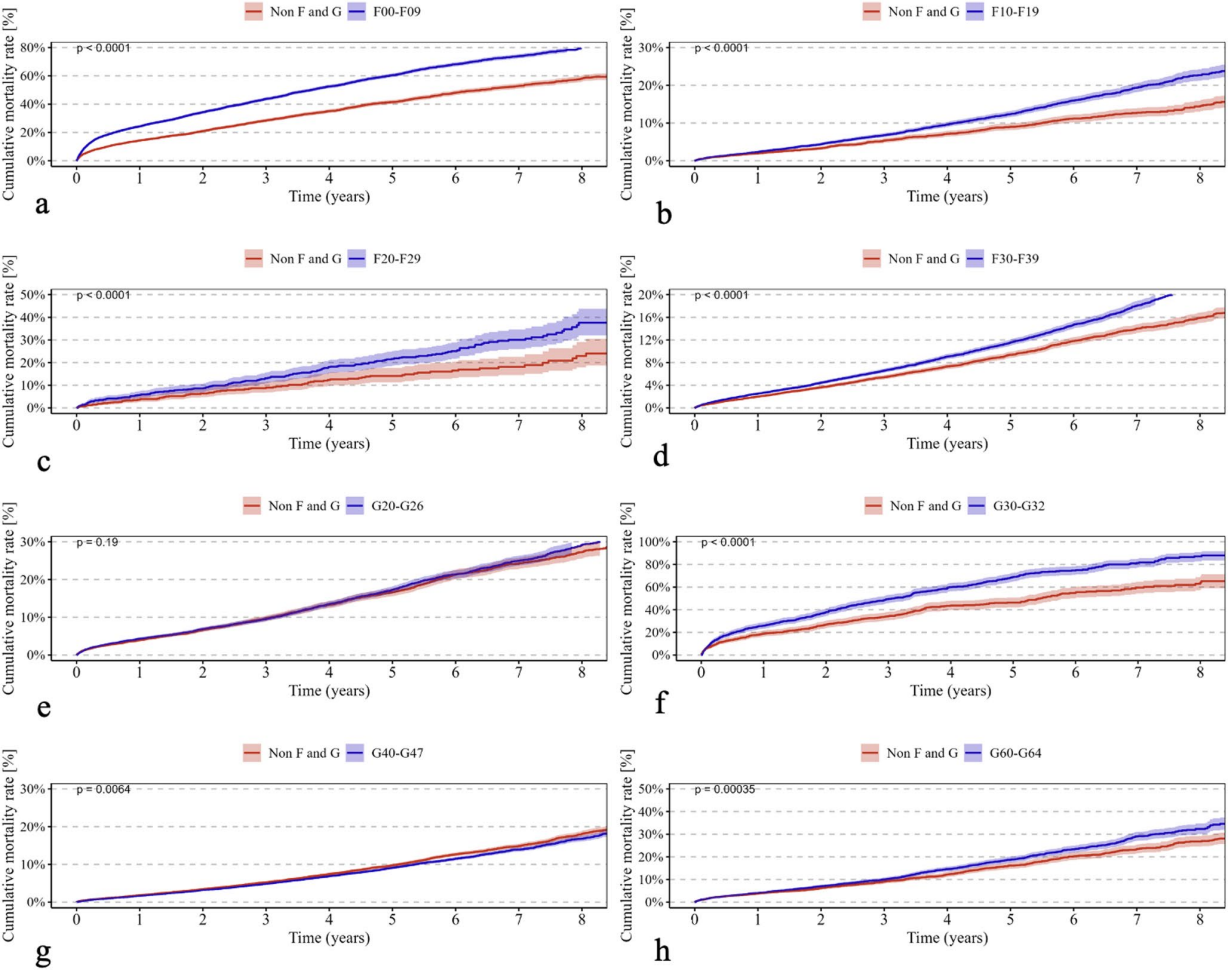
Shows Kaplan–Meier curves (**a**–**h**) illustrating the cumulative incidence of mortality over an eight-year observation period in patients with the following ICD-10 diagnoses (red curves) compared with a control group (blue curves): (**a**) F00–F09 (“Organic, including symptomatic, mental disorders,” *p* < 0.0001), (**b**) F10–F19 (“Mental and Behavioral Disorders due to Psychoactive Substance Use,” *p* < 0.0001), (**c**) F20–F29 (“Schizophrenia, schizotypal, and delusional disorders,” *p* < 0.0001), (**d**) F30–F39 (“Affective Disorders,” *p* < 0.0001), (**e**) G20–G26 (“Extrapyramidal and Movement Disorders,” *p* = 0.19), (**f**) G30–G32 (“Degenerative diseases of the nervous system,” *p* < 0.0001), (**g**) G40–G47 (“Episodic and paroxysmal disorders,” *p* = 0.0064), and (**h**) G60–G64 (“Polyneuropathies and other disorders of the peripheral nervous system,” *p* = 0.00035). The *p*-values indicate the significance of between-group differences

**Fig. 3:**
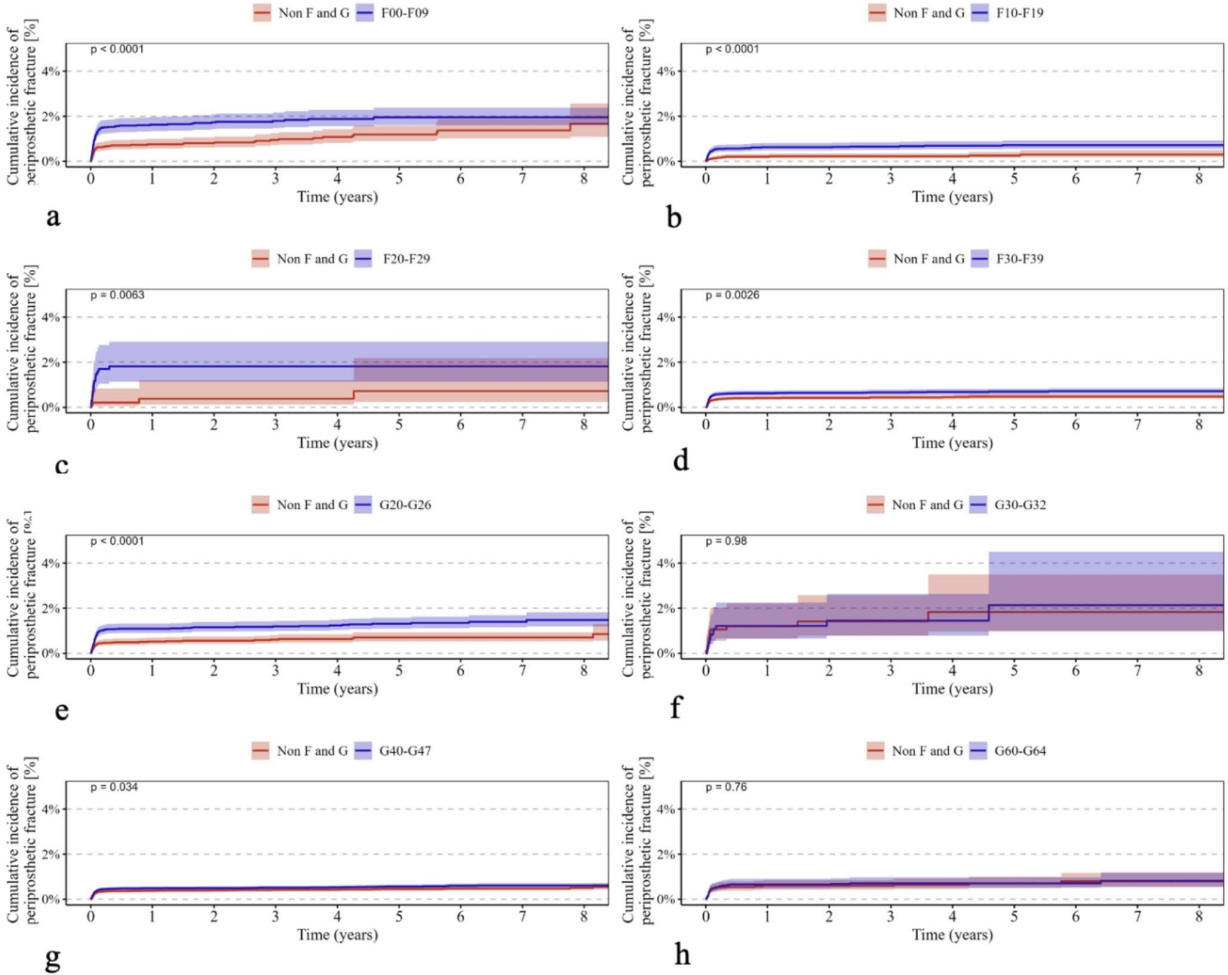
Shows Kaplan–Meier curves (**a**–**h**) illustrating the cumulative incidence of periprosthetic fractures over an eight-year observation period in patients with the following ICD-10 diagnoses (red curves) compared with a control group (blue curves): (**a**) F00–F09 (“Organic, including symptomatic, mental disorders,” *p* < 0.0001), (**b**) F10–F19 (“Mental and Behavioral Disorders due to Psychoactive Substance Use,” *p* < 0.0001), (**c**) F20–F29 (“Schizophrenia, schizotypal, and delusional disorders,” *p* = 0.0063), (**d**) F30–F39 (“Affective Disorders,” *p* = 0.0026), (**e**) G20–G26 (“Extrapyramidal and Movement Disorders,” *p* < 0.0001), (**f**) G30–G32 (“Degenerative diseases of the nervous system,” *p* = 0.98), (**g**) G40–G47 (“Episodic and paroxysmal disorders,” *p* = 0.034), and (**h**) G60–G64 (“Polyneuropathies and other disorders of the peripheral nervous system,” *p* = 0.76). The *p*-values indicate the significance of between-group differences

**Fig. 4:**
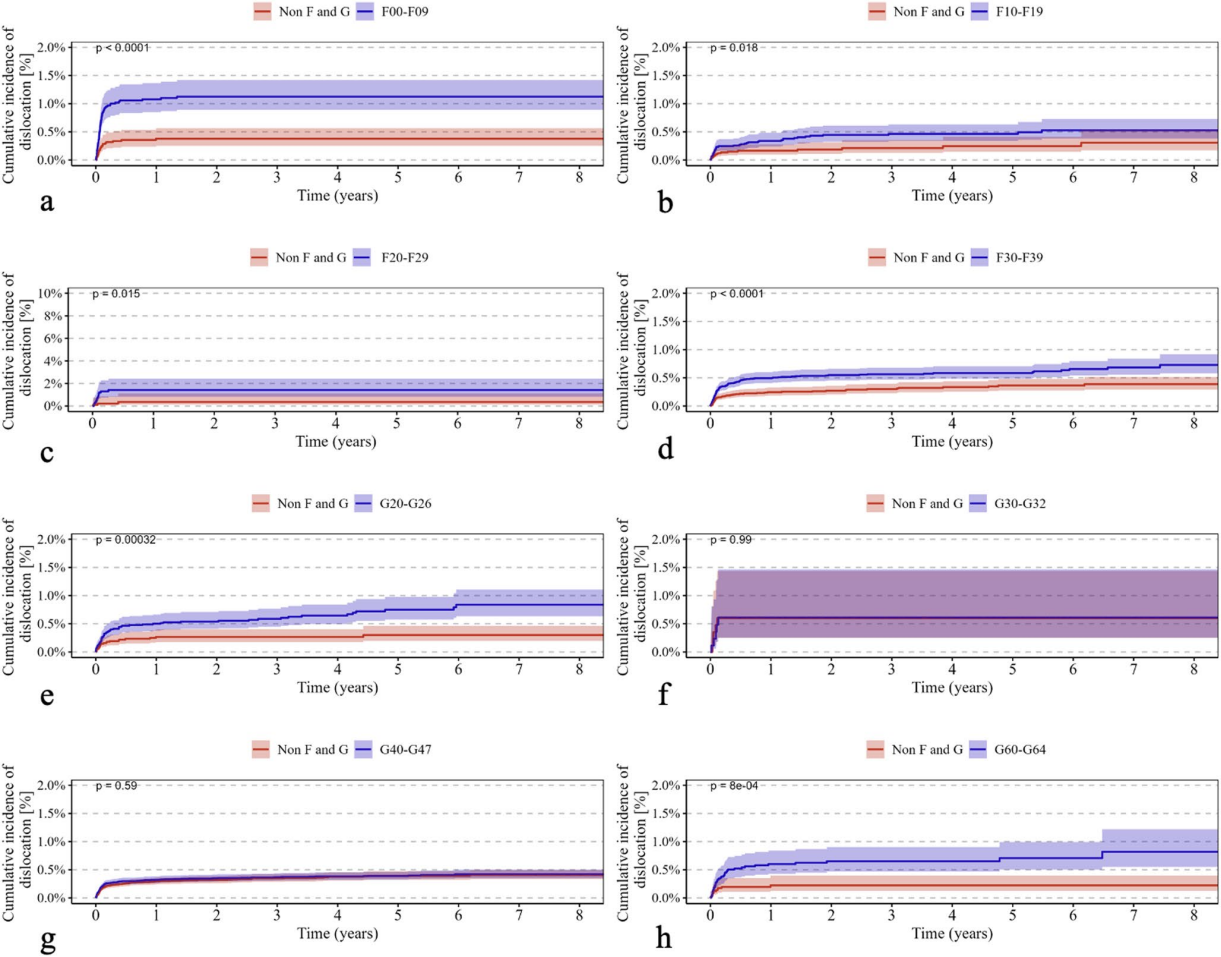
Shows Kaplan–Meier curves (**a**–**h**) illustrating the cumulative incidence of dislocation over an eight-year observation period in patients with the following ICD-10 diagnoses (red curves) compared with a control group (blue curves): (**a**) F00–F09 (“Organic, including symptomatic, mental disorders,” *p* < 0.0001), (**b**) F10–F19 (“Mental and Behavioral Disorders due to Psychoactive Substance Use,” *p* = 0.018), (**c**) F20–F29 (“Schizophrenia, schizotypal, and delusional disorders,” *p* = 0.015), (**d**) F30–F39 (“Affective Disorders,” *p* < 0.0001), (**e**) G20–G26 (“Extrapyramidal and Movement Disorders,” *p* = 0.00032), (**f**) G30–G32 (“Degenerative diseases of the nervous system,” *p* = 0.99), (**g**) G40–G47 (“Episodic and paroxysmal disorders,” *p* = 0.59), and (**h**) G60–G64 (“Polyneuropathies and other disorders of the peripheral nervous system,” *p* = 0.0008). The *p*-values indicate the significance of between-group differences

**Fig. 5:**
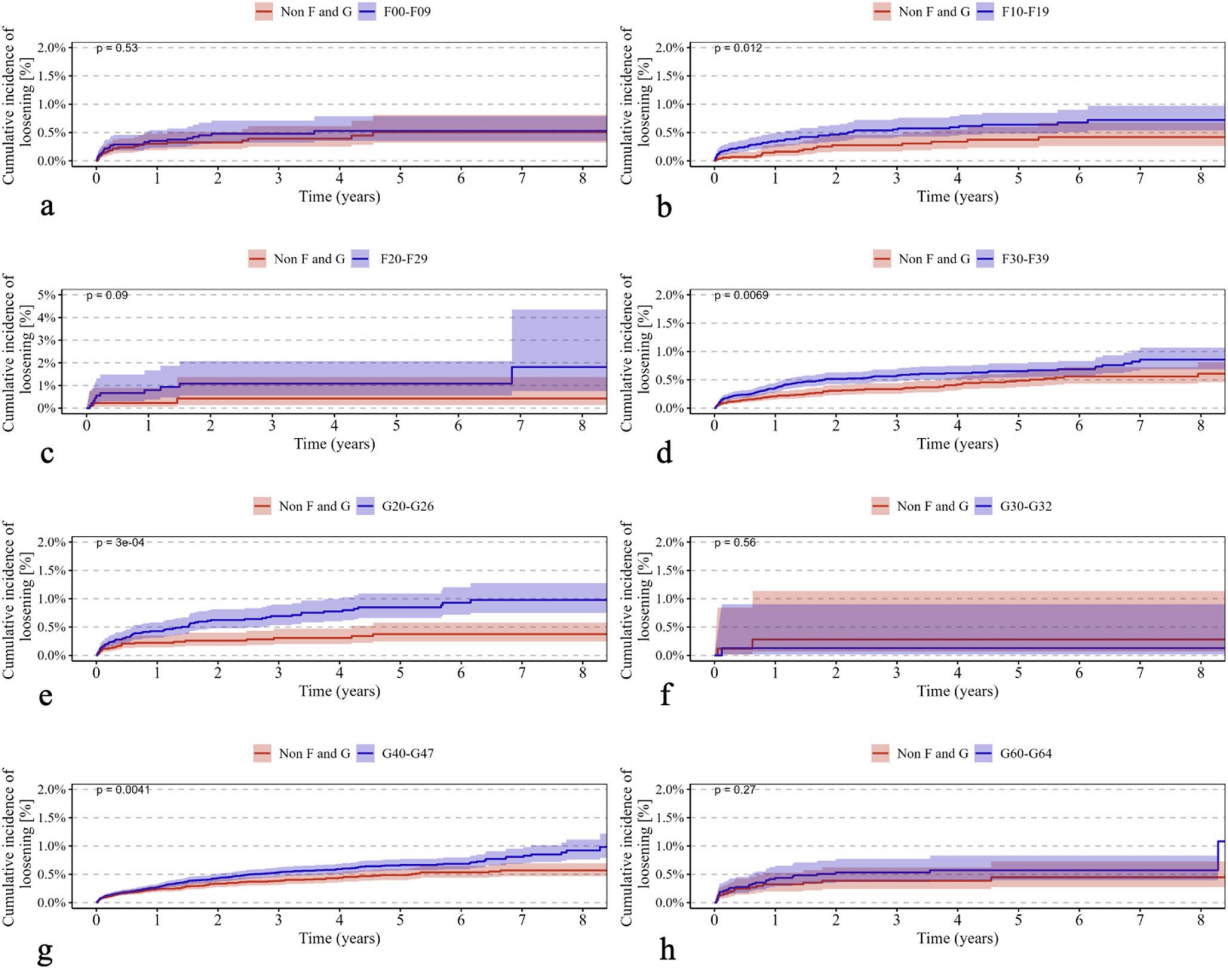
Shows Kaplan–Meier curves (a–h) illustrating the cumulative incidence of loosening over an eight-year observation period in patients with the following ICD-10 diagnoses (red curves) compared with a control group (blue curves): (**a**) F00–F09 (“Organic, including symptomatic, mental disorders,” *p* = 0.53), (**b**) F10–F19 (“Mental and Behavioral Disorders due to Psychoactive Substance Use,” *p* = 0.012), (**c**) F20–F29 (“Schizophrenia, schizotypal, and delusional disorders,” *p* = 0.09), (**d**) F30–F39 (“Affective Disorders,” *p* = 0.0069), (**e**) G20–G26 (“Extrapyramidal and Movement Disorders,” *p* = 0.0003, (**f**) G30–G32 (“Degenerative diseases of the nervous system,” *p* = 0.56), (**g**) G40–G47 (“Episodic and paroxysmal disorders,” *p* = 0.0041), and (**h**) G60–G64 (“Polyneuropathies and other disorders of the peripheral nervous system,” *p* = 0.27). The *p*-values indicate the significance of between-group differences

Psychiatric disorders classified under ICD-10 F00–F09 were associated with a significantly higher cumulative revision rate (*p* < 0.0001) (Fig. 1a) and increased all-cause mortality (*p* < 0.0001) (Fig. 2a). In addition, the incidence of periprosthetic fractures (*p* < 0.0001) (Fig. 3a) and dislocations (*p* < 0.0001) (Fig. 4a) was significantly elevated, while no significant difference was observed for implant loosening (*p* = 0.53) (Fig. 5a).

For psychiatric disorders according to ICD-10 F10–F19, both revision rates (*p* < 0.0001) (Fig. 1b) and mortality (*p* < 0.0001) (Fig. 2b) were significantly higher. The risk of periprosthetic fractures (*p* < 0.0001) (Fig. 3b), dislocations (*p* = 0.018) (Fig. 4b), and loosening (*p* = 0.012) (Fig. 5b) was also significantly increased (Table 1b).

Psychiatric disorders classified as ICD-10 F20–F29 were associated with a significantly elevated revision rate (*p* < 0.0001) (Fig. 1c) and mortality (*p* < 0.0001) (Fig. 2c). Periprosthetic fractures (*p* = 0.0063) (Fig. 3c) and dislocations (*p* = 0.015) (Fig. 4c) occurred more frequently, whereas implant loosening showed no statistically significant increase (*p* = 0.09) (Fig. 5c).

Psychiatric disorders under ICD-10 F30–F39 were similarly associated with significantly higher rates of revision (*p* < 0.0001) (Fig. 1d) and mortality (*p* < 0.0001) (Fig. 2d). Moreover, increased incidences of periprosthetic fractures (*p* = 0.0026) (Fig. 3d), dislocations (*p* < 0.0001) (Fig. 4d), and implant loosening (*p* = 0.0069) (Fig. 5d) were observed (Table 2).

**Table 2:** Distribution of revision indications for hip arthroplasty in patients without and with psychiatric disorders (ICD-10 F00–F09; F10-F19; F20-F29; F30-F39; G20–G26; G30-G32; G40-G47; G60-G64)

	F00-F09		F10-F19	F20-F29	F30-F39	G20–G26	G30–G32	G40–G47	G60–G64
	Without psychiatric *N* = 363/ With psychiatric *N* = 602	Without psychiatric *N* = 245/ With psychiatric *N* = 468		Without psychiatric *N* = 30/ With psychiatric *N* = 80	Without psychiatric *N* = 703/ With psychiatric *N* = 1,138	Without neurological *N* = 383/ With neurological *N* = 592	Without neurological *N* = 42/ With neurological *N* = 63	Without neurological *N* = 1,344/ With neurological *N* = 1,715	Without neurological *N* = 213/ With neurological *N* = 378
Reason for Revision									
Component failure	3 (1.3%)/ 1 (0.3%)	1 (0.6%)/ 5 (1.5%)		0 (0%)/1 (1.6%)	4 (0.8%)/9 (1.1%)	1 (0.4%)/4 (0.9%)	1 (3.3%)/0 (0%)	6 (0.7%)/5 (0.4%)	2 (1.3%)/2 (0.8%)
Condition after removal	5 (2.1%)/ 11 (3.0%)	9 (5.3%)/ 8 (2.5%)		0 (0%)/1 (1.6%)	19 (4.0%)/21 (2.7%)	8 (3.3%)/6 (1.4%)	2 (6.7%)/1 (2.7%)	28 (3.1%)/32 (2.7%)	5 (3.3%)/5 (2.1%)
Dislocation	24 (10%)/ 70 (19%)	18 (11%)/ 42 (13%)		3 (18%)/13 (21%)	59 (12%)/120 (15%)	24 (10%)/63 (14%)	5 (17%)/5 (14%)	118 (13%)/138 (12%)	12 (8.0%)/38 (16%)
Infection	85 (36%)/ 117 (31%)	77 (46%)/ 95 (29%)		6 (35%)/17 (27%)	179 (37%)/266 (34%)	91 (38%)/111 (25%)	6 (20%)/14 (38%)	343 (38%)/428 (36%)	58 (39%)/84 (35%)
Loosening	23 (9.8%)/ 27 (7.3%)	20 (12%)/ 52 (16%)		3 (18%)/10 (16%)	69 (14%)/120 (15%)	25 (10%)/69 (16%)	2 (6.7%)/1 (2.7%)	125 (14%)/207 (18%)	19 (13%)/30 (12%)
Malalignment	3 (1.3%)/ 5 (1.3%)	6 (3.6%)/ 8 (2.5%)		0 (0%)/0 (0%)	15 (3.1%)/22 (2.8%)	3 (1.3%)/7 (1.6%)	0 (0%)/0 (0%)	18 (2.0%)/28 (2.4%)	3 (2.0%)/7 (2.9%)
Osteolysis	0 (0%)/ 1 (0.3%)	1 (0.6%)/ 0 (0%)		0 (0%)/0 (0%)	1 (0.2%)/2 (0.3%)	2 (0.8%)/1 (0.2%)	0 (0%)/1 (2.7%)	2 (0.2%)/2 (0.2%)	2 (1.3%)/0 (0%)
Other reasons	26 (11%)/ 26 (7.0%)	15 (8.9%)/ 46 (14%)		1 (5.9%)/4 (6.3%)	39 (8.1%)/77 (9.8%)	28 (12%)/49 (11%)	2 (6.7%)/3 (8.1%)	89 (10.0%)/128 (11%)	13 (8.7%)/32 (13%)
Periprosthetic fracture	63 (27%)/ 113 (30%)	22 (13%)/ 63 (20%)		4 (24%)/17 (27%)	91 (19%)/140 (18%)	56 (23%)/122 (28%)	12 (40%)/12 (32%)	154 (17%)/201 (17%)	36 (24%)/41 (17%)
Progression of arthrosis	2 (0.9%)/ 0 (0%)	0 (0%)/ 0 (0%)		0 (0%)/0 (0%)	1 (0.2%)/0 (0%)	0 (0%)/1 (0.2%)	0 (0%)/0 (0%)	0 (0%)/0 (0%)	0 (0%)/0 (0%)
Wear	1 (0.4%)/ 1 (0.3%)	0 (0%)/ 4 (1.2%)		0 (0%)/0 (0%)	3 (0.6%)/10 (1.3%)	2 (0.8%)/5 (1.1%)	0 (0%)/0 (0%)	8 (0.9%)/10 (0.8%)	0 (0%)/3 (1.2%)
Unknown	128/ 230	76/ 145		13/17	223/351	143/154	12/26	453/536	63/136

Distribution of revision indications in patients without and with psychiatric pre-existing conditions across ICD-10 diagnostic groups (F00–F09, F10–F19, F20–F29, F30–F39, G20–G26, G30–G32, G40–G47, G60–G64). The most common reasons for revision included infection, periprosthetic fracture, dislocation, and loosening. Numbers are presented as *n* (%) for each indication

Among neurological disorders classified under ICD-10 G20–G26, the cumulative revision rate was significantly higher (*p* < 0.0001) (Fig. 1e). Dislocations (*p* = 0.00032) (Fig. 4e) and implant loosening (*p* = 0.0003) (Fig. 5e) were also more frequent. Mortality did not differ significantly from controls (*p* = 0.19) (Fig. 2e). Periprosthetic fractures were more common (*p* < 0.0001) (Fig. 3e).

Neurological disorders according to ICD-10 G30–G32 were associated with a significantly increased mortality rate (*p* < 0.0001) (Fig. 2f) and a moderately but significantly elevated revision rate (*p* = 0.04) (Fig. 1f). No significant differences were found for periprosthetic fractures (*p* = 0.98) (Fig. 3f), dislocations (*p* = 0.99) (Fig. 4f), or loosening (*p* = 0.56) (Fig. 5f).

In patients with neurological disorders under ICD-10 G40–G47, revision (*p* < 0.0001) (Fig. 1g), mortality (*p* = 0.0064) (Fig. 2g), periprosthetic fractures (*p* = 0.034) (Fig. 3g), and implant loosening (*p* = 0.0041) (Fig. 5g) were significantly more frequent. Dislocations did not differ significantly (*p* = 0.59) (Fig. 4g).

Neurological disorders classified under ICD-10 G60–G64 were associated with significantly higher rates of revision (*p* < 0.0001) (Fig. 1h), mortality (*p* = 0.00035) (Fig. 2h), and dislocations (*p* = 0.0008) (Fig. 4h). No significant differences were observed for periprosthetic fractures (*p* = 0.76) (Fig. 3h) or implant loosening (*p* = 0.27) (Fig. 5h).

## Discussion

Our registry-based analysis of 190,340 primary cementless hip endoprostheses is the first study to investigate the impact of neurological and psychiatric pre-existing conditions on postoperative outcomes using a comprehensive, ICD 10-based approach. We demonstrated that several subgroups (F00–F09, F10–F19, F20–F29, F30–F39, G20–G26, G30–G32, G40–G47, and G60–G64) exhibit significantly increased revision and mortality rates. These findings underscore the substantial influence of psychiatric and neurological diagnoses on postoperative risk following hip arthroplasty and emphasize the need to account for these conditions in preoperative risk stratification.

### Limitations

Like all registry-based investigations, our study has certain constraints. First, it may be susceptible to incomplete or inaccurate coding, given that the data originate from routine documentation. Second, no detailed clinical information was available on the severity of each neurological or psychiatric disorder, nor on relevant therapies or socioeconomic factors. Third, missing or non-specific information in BMI and revision reasons (“not specified/unknown”) could have introduced bias in specific analyses. Nevertheless, our extensive multicenter dataset, supported by standardized documentation, reflects real-world clinical practice and thus provides valuable insights.

Among 7,166 matched patients (vs. 7,166 controls) in the F00–F09 (Organic, Including Symptomatic, Mental Disorders) subgroup, we identified significantly higher revision and mortality rates, along with more frequent dislocations and periprosthetic fractures. These outcomes are consistent with Zhang et al., who reported increased dislocation (OR 1.87), fracture (OR 1.29), and revision (OR 1.23) rates in 44,536 dementia patients following hemiarthroplasty [6]. Importantly, Zhang et al. only tracked one-year outcomes, whereas our findings extend to an eight-year period. Hernandez et al. also noted elevated revision (OR 1.71), delirium (OR 5.53), and a greater need for care facilities (OR 2.31) in demented THA patients [7]. By contrast, Chandrupatla et al. observed higher revision rates in THA patients with diabetes, a frequent dementia comorbidity, yet no consistent rise in dislocations or fractures [8].

Within the F10–F19 (Mental and Behavioral Disorders Due to Psychoactive Substances) subgroup, we detected significantly increased mortality in tandem with higher rates of revision, fracture, and dislocation. Best et al. likewise documented a heightened risk of surgical complications (OR 1.29), including these outcomes, as well as elevated mortality in alcohol-dependent patients [9]. Interestingly, Kamalapathy et al. found only a moderate risk increase for outpatient procedures, suggesting that careful patient selection can reduce complications [10]. Such a discrepancy underscores the role of clinical context, as our registry reflects routine inpatient care, typically involving more complex cases.

Patients in the F20–F29 (Schizophrenia, Schizotypal, and Delusional Disorders) subgroup experienced significantly increased rates of revision, fracture, dislocation, and mortality. Gholson et al. likewise detected greater mechanical implant failure (OR 3.2), transfusions (OR 2.4), and prolonged hospitalization in affected individuals [11]. Nevertheless, Gholson’s propensity score–matched data did not indicate elevated periprosthetic joint infection (PJI) or dislocation, which may point to differences in clinical management strategies [11]. Our results, however, demonstrate that these risks remain high over a longer observation period, emphasizing the need for sustained postoperative surveillance.

Within the F30–F39 (Affective Disorders) subgroup, significantly increased mortality was accompanied by higher incidences of fracture, dislocation, and revision. Wilson et al. corroborated these findings by showing raised rates of PJI (OR 1.52), sepsis (OR 1.86), and revision (OR 1.36) in depressed patients post-revision THA [12]. In contrast, Kamalapathy et al. reported no substantial escalation in revision or complication rates among outpatients with affective disorders, suggesting a possibly fitter cohort [10]. Our data, drawn from an inpatient registry, likely capture a broader real-world population.

Notably higher rates of revision, dislocation, fracture, and loosening emerged in the G20–G26 (Extrapyramidal and Movement Disorders) subgroup, particularly among those with Parkinson’s disease. These trends mirror findings by Holzapfel et al., who observed a significantly elevated revision rate (OR 5.08) [4], and Newman et al., who reported a 54% uptick in overall complications [3]. Additionally, Rong et al. described worsening functional outcomes at advanced Hoehn–Yahr stages [13]. Meanwhile, McCormack et al.’s systematic review identified moderate revision rates (about 12.8%) yet significant functional limitations, even with dual mobility cups [14]. Discrepancies likely reflect varying study priorities, surgical complications versus functional endpoints.

A markedly higher mortality rate was apparent in the G30–G32 (Degenerative Diseases of the Nervous System) subgroup, although fractures, dislocations, and loosening did not increase significantly. Fontalis et al. and Rong et al. reported similar findings of elevated mortality and revision rates but inconclusive data on mechanical complications [13, 15]. Chandrupatla et al. also found no notable difference in dislocation rates among THA patients with avascular necrosis or related degenerative disorders [8]. Although restricted mobility may contribute to higher mortality, it may simultaneously guard against certain mechanical failures.

Our data indicate elevated revision, fracture, and loosening rates in the G40–G47 (Episodic and Paroxysmal Disorders) subgroup, including individuals with epilepsy or comparable conditions. Cole et al. likewise observed heightened fracture (OR 2.39), dislocation (OR 1.54), and loosening (OR 1.40) rates in epilepsy patients [16]. By comparison, Couch et al. noted increased perioperative morbidity (pneumonia, delirium, mechanical ventilation) but no discernible rise in revision surgeries [17]. Such contrasts may stem from differences in follow-up period, epilepsy severity, or analytical design.

A significantly higher dislocation rate and mortality were evident in the G60–G64 (Polyneuropathies and Other Peripheral Nerve Disorders) subgroup, whereas fractures and loosening were not substantially elevated. Although direct comparisons are limited, Cole et al. reported increased mechanical complications in neurologically impaired patients, likely owing to fall risk and muscular discoordination [16]. Our results thus provide essential new insight into a clinically relevant yet relatively unstudied population.

The present findings suggest that patients with pre-existing neurological or psychiatric conditions have a distinct risk profile following hip arthroplasty, which is not yet systematically considered in routine clinical practice. Given the increased revision and mortality rates observed in subgroups such as F00–F09 (e.g., dementia) and G60–G64 (polyneuropathy), these diagnoses should be explicitly incorporated into preoperative risk assessment and patient counseling. The use of tailored implant designs, intensive early mobilization, or geriatric-psychiatric co-management strategies may lead to improved outcomes. Current guidelines offer few specific recommendations on these issues, underscoring the urgent need for a systematic derivation of dedicated care pathways based on the available data.

## Conclusion

Neurological and psychiatric pre-existing conditions differentially affect risk after hip arthroplasty. F00–F09 was linked to higher mortality, dislocations, and fractures; F10–F19 to significantly increased complication rates; and F20–F29 and F30–F39 to more frequent mechanical failures and elevated mortality. G20–G26 primarily led to dislocations, G30–G32 to higher mortality without additional mechanical risks, G40–G47 exhibited increased loosening and fracture rates, and G60–G64 showed a pronounced tendency toward instability and higher mortality. These distinct risk profiles are clinically relevant for both preoperative evaluation and postoperative care.

## Data Availability

No datasets were generated or analysed during the current study.
